# Quality of Life Post Lympho-Venous Anastomosis in Breast Cancer–Related Lymphedema in the Indian Population

**DOI:** 10.1055/s-0045-1806746

**Published:** 2025-03-18

**Authors:** Annika Marwah, Ashok Basur Chandrappa, Srikanth Vasudevan, Ananteshwar Y.N, Aditya Jana, Jeet Radadia, Pooja Shetty, Serena B.

**Affiliations:** 1 Department of Plastic and Reconstructive Surgery, Manipal hospital, Bangalore, India; 2 Department of General Surgery, Manipal Hospital, Bangalore

**Keywords:** lymphedema, breast cancer–related lymphedema, indocyanine green, lympho-venous anastomosis, Indian limb lymphedema scoring system

## Abstract

**Introduction:**

Secondary lymphedema negatively impacts the quality of life. Lympho-venous anastomosis (LVA) has shown to attenuate lymphedema symptoms.

**Objective:**

The aim of the study is to evaluate the efficacy, patient satisfaction, and quality of life following LVA for breast cancer–related lymphedema (BCRL) in the Indian setup.

**Materials and Methods:**

We conducted a prospective, nonrandomized, feasibility study at a single institute. Sixteen patients with BCRL undergoing secondary LVA between May 2020 and December 2021 were included in the study. Volumetry was done preoperatively and then 6, 12, and 18 months postoperatively. The Indian limb lymphedema scoring (ILLS) system questionnaire and satisfaction scoring were done 1 year postoperatively.

**Results:**

Sixteen patients undergoing secondary LVA for BCRL were included in the study. The preoperative mean difference in limb circumference volume was 804.41 ± 472.01. This was reduced to 471.81 ± 292.216 mL at the 6-month follow-up ( *t* = –6.6323; *p* ≤ 0.00001), 448.58 ± 255.93 ( *t* = –5.5295; *p* = 0.00006) at 12 months, and 445.25 ± 345.78 ( *t* = –6.8957; *p* ≤ 0.00001) at 18 months of follow-up. The mean volume difference between the two limbs at 12 months post-LVA was 515.3144 ± 284.2007 mL ( *t* = 1.9972; *p* = 0.1250) and 362.7957 ± 201.9709 mL ( *t* = 0.1221; *p* = 0.4522). The number of LVAs did not show a statistically significant difference in outcome with a volume difference of 515.3144 ± 284.2007 and 362.7957 ± 201.9709 mL in groups with less than four and more than four anastomosis and a *p* -value of 0.1250 and 0.4522, respectively. Similarly, body mass index (BMI) and duration of lymphedema did not show a statistically significant difference in outcome ( *p* = 0.2648 and 0.2281, respectively). The mean total ILLS were 31.35 and 13.66 pre- and postoperatively, respectively, showing a statistically significant improvement in the quality of life, with a *p* -value of 0.00023.

**Conclusion:**

LVA significantly improves the quality of life and reduces limb volume, with stabilization occurring at 18 months, and the number of anastomoses, patient's BMI, and lymphedema duration did not affect volume reduction in our set of patients.

## Introduction

Lymphedema is characterized by excessive accumulation of protein-rich fluid in the interstitium. 1 Mastectomy/breast-conserving surgery with axillary lymph node dissection (ALND) is offered as treatment for certain breast cancer patients for improving the survival rate. But removal of the axillary lymph nodes may also disrupt the lymphatic circulation of the upper limb, increasing the risk of developing lymphedema. 2

Symptoms of lymphedema include swelling, heaviness, and tightness of extremities, along with pain and paresthesia, with increased circumference being a prominent sign. 2 3 Lymphedema is debilitating and negatively impacts the quality of life in the physical, psychological, and social domains. 4 5 6

The surgical treatment of limb lymphedema includes radical resections for advanced stages and newer microsurgical techniques like lympho-venous anastomoses (LVAs), lymph node to venous anastomoses, vascularized lymph node transfer (VLNT), and vascularized lymph vessel transfer. 7 Experimental and clinical anatomical studies suggested that lymphatics with preserved contractility should be utilized for LVA to enhance the long-term effect of surgery. 8 Furthermore, lymph pressure and flow measurements showed that lympho-venous shunts are successful if performed in patients with segmental obstruction of proximal lymphatics, given good quality of distal lymphatics. 9

Microsurgical techniques like LVA have shown long-term patency, volume reduction, and mitigate lymphedema symptoms.

## Objectives

The aim of the study is to evaluate following parameters following secondary LVA of the upper limb post ALND in an Indian setup in a tertiary hospital:

- Efficacy of secondary LVA based on volume reduction.
- Quality of life.
- Patient satisfaction.

## Materials and Methods

We conducted a prospective, nonrandomized, feasibility study at a single institute (Manipal Hospitals, Old Airport Road, Bangalore). All patients between the ages of 30 and 65 years who met the eligibility criteria and consulted the Department of Plastic and Reconstructive Surgery between May 2020 and December 2021 were included in the study. All the patients had prior ALND with radiation therapy. The minimum follow-up period was about 18 months. Patients were assessed with volumetric analysis at 6, 12, and 18 months following surgery and quality-of-life survey done preoperatively and at 12 months postoperatively. The results were analyzed by deductive thematic analysis and paired *t* -test was used to compare the pre- and postoperative data, satisfaction scoring was done at 12 months postoperatively, and the qualitative data were analyzed by mean.

The eligibility criteria were the following:

- Patients aged between 30 and 65 years with breast cancer–related lymphedema (BCRL) consenting for LVA.
- More than 10% difference in volume by volumetry.
- Stage 2 and early stage 3 lymphedema on Indocyanine green (ICG) study with linear channels and splash/stardust pattern.

The exclusion criteria were the following:

- Infective cause of lymphedema.
- Late stage 3 and 4 lymphedema.
- Patients undergoing additional VLNT or debulking procedure.
- Presence of comorbidities that can cause limb edema.
- Inability to answer the questionnaire.

### Diagnosis and Grading

After thorough history-taking and examination of all the patients, volumetry was done by measuring the limb circumference using a measuring tape, and the volume of the limb was calculated using a computer-based formula considering the limb as a frustum.

All patients having more than 10% difference in the volume between the two limbs underwent ICG lymphography by injecting 1 mL of the ICG dye intradermally on three points (second, fourth web spaces, and medial to palmaris longus tendon) in the upper limb. The dye is visualized using an infrared camera (750–800 nm) to look for dermal backflow patterns right after injection and 5, 20, and 60 minutes postinjection, that helped in staging of lymphedema and the line of management.

**Fig. 1:**
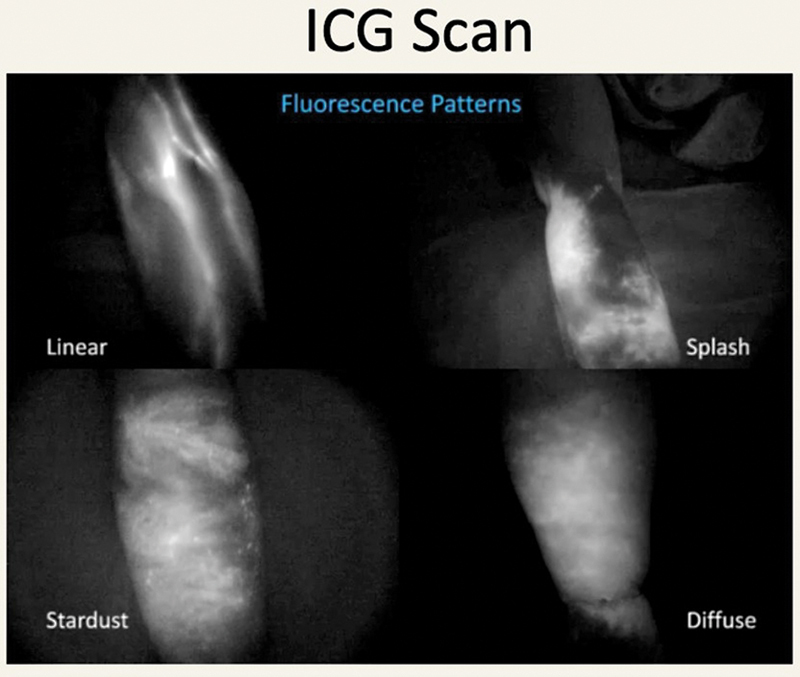
Various patterns of limb lymphedema on indocyanine green (ICG) scan.

In ICG stage 2 (linear channels with splash) and early stage 3 (stardusting) lymphedema, 10 patients were offered class 2 compression garments for 3 months to check for their compliance, and patients who did not improve were subjected to LVA ( Fig. 1 ).

### Surgical Technique

After a preoperative workup, assessment of anesthesia fitness, and obtaining the patient's consent, surgery was carried out under local anesthesia (LA)/general anesthesia (GA).

**Fig. 2:**
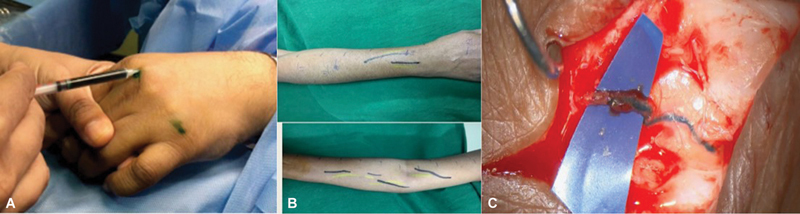
Surgical technique. ( **A** ) Injecting indocyanine green (ICG) dye and methylene blue. ( **B** ) Marking of linear channels by ICG lymphography and superficial visible veins. ( **C** ) Stained lymphatic (methylene blue), explored adjacent vein.

**Fig. 3:**
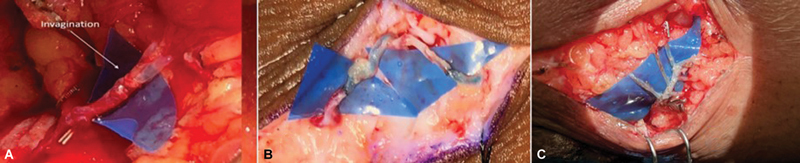
Types of lympho-venous anastomoses (LVAs). ( **A** ) Octopus technique. ( **B** ) Two end-to-end anastomosis. ( **C** ) Multiple end-to-side anastomosis.

The prerecorded ICG scan was checked, or it was repeated after induction to map the linear lymphatic channels on the limb followed by injection of 1 mL of methylene blue on the same points, which helped us to identify the blue stained lymphatics visually under magnification much easily. At the same time, any visible superficial veins were also marked. Linear incisions of 3 to 4 cm were made to expose the marked lymphatics under an operating microscope, which took up the blue dye, and an adjacent superficial vein was also explored ( Fig. 2 ). Depending on the quality of the lymphatic, caliber, and size discrepancy with the neighbouring vein, anastomosis was carried out in an end-to-end, end-to-side, or invagination manner using 10–0/11–0 Ethilon sutures ( Fig. 3 ). In the cases with multiple small lymphatics, the octopus technique was used for LVA. We performed an on-table ICG scan after anastomosis to confirm the patency of the same. Incisions were closed using 5–0 Prolene sutures followed by the application of a compression dressing and limb elevation in the postoperative period.

The first dressing was changed on the second/third postoperative day and a short stretch bandage was used thereafter till suture removal at 10 days. Class 2 compression garment was given for 6 to 12 months postprocedure.

The patients were followed up with volumetry scans at 6, 12, and 18 months, and patient satisfaction and quality of life survey was done at 12 months postsurgery.

### Data Collection

Data were collected during patients' clinical visits at the outpatient department. When potential participants met the inclusion criteria, they were given verbal information about the study and asked to provide written informed consent before participation. The participants completed the questionnaire given in a private clinical room, and their limb circumferences were measured. After that, the participants' medical records were reviewed to obtain their health and medical information.

### Statistical Analysis

All continuous variables are represented by an average percentage. The paired *t* -test was used to evaluate the pre- and postoperative changes.

## Questionnaire

We used a questionnaire to assess the quality of life of Indian patients after secondary LVA after mastectomy/ALND. Lymphedema-related disabilities were identified in two ways. First, through a review of literature through PubMed, wherein a bibliography was looked at to search for other relevant articles. Furthermore, 16 patients included in our study were evaluated in one-on-one interviews regarding their functional and social status, their disability, and problems they are facing, which they attributed to limb lymphedema. They were also interviewed regarding their cultural norms and beliefs. All participants were subjected to the following open-ended questions to understand their problems and disability better:

- Which complaints do you have related to lymphedema?
- Which adverse feelings do you have related to lymphedema?
- Which activities are difficult or not possible to perform because of your lymphedema?
- Do you have any other remarks?
- What are your cultural norms and beliefs?

The data collected included causes of secondary lymphedema (treatment of breast carcinoma, malignant phyllodes), side of limb affected, duration of lymphedema (in months), and current medical treatment of lymphedema (manual lymph drainage, layered bandage or compression garment, exercises, or skin care). Patients' characteristic and demographic data were collected by reviewing their medical records. Lymphedema was quantified by volumetry.

Based on the information derived through a review of the literature and interviewing the study subjects, the Indian limb lymphedema scoring (ILLS) system was devised, which was specifically developed for the Indian population while keeping their cultural practices in mind. The questionnaire was proposed in four domains, namely, physical well-being, mental well-being, social well-being, and activities of daily living with a total of 10 parameters. Each parameter was scored between 0 and 5 (0 = not at all; 1 = minimal; 2 = mild; 3 = moderate; 4 = severe; 5 = very severe), and a total score was given from 0 to 50.

The questionnaire describes disability, activity restrictions, and social limitations in the Indian population of upper lymphedema in detail.

All the patients (16/16) completed the follow-up and survey questionnaire, giving a response rate of 100%.

## Results

Between May 2020 and December 2021, 16 consecutive patients with secondary lymphedema post-ALND were recruited from our department. The patients are followed up for a minimum period of 18 months postoperatively.

The total number of patients included in the study was 16 ( Table 1 ), with an average age of 56.3 years, and body mass index (BMI) of 30.05 kg/m ^2^ . The majority of the sample population were married (87.5%) and more than half of them were employed. In all, 81.25% patients had a primary diagnosis of breast cancer and 18.75% had malignant phyllodes. The average duration for the presence of lymphedema was 19.66 ± 4.67 months. The average duration of surgery was 264.37 ± 19.51 minutes.

**Table 1:** demographics and patient characteristics

Characteristics	*N*	Percentage/mean
Age	16	56.3 ± 5.6 y
Marital status: married	14	87.5
Employment	9	56
BMI	–	30.05 ± 4.89 kg/m ^2^
Primary diagnosis: breast cancer	13	81.25
Malignant phyllodes	3	18.75
Duration of lymphedema	–	19.66 ± 4.67 mo
Average duration of surgery		264.37 ± 19.51 min
No. of anastomosis	1–8	4.68 ± 2.65

The average number of anastomoses was 4.68 ± 2.65. There were nine patients with less than four anastomosis and seven patients had more than four anastomoses. The mean volume difference at 12 months post-LVA was 515.3144 ± 284.2007 and 362.7957 ± 201.9709 mL ( *t* = 1.9972) in each group with a percentage volume reduction of 15.89 and 15.37%. We did not find any statistically significant difference in volume reductions ( Table 2 ) from baseline at 12 months of follow-up between the two groups ( *p* = 0.1250 and 0.4522 for volume and percentage).

**Table 2:** Relation between the number of anastomosis and volume reductions at 12 months after lympho-venous anastomosis (LVA)

No. of anastomosis	Volume difference at 12 mo (mL)	Volume difference at 12 mo (%)
≤4	515.3144 ± 284.2007	15.89556 ± 7.7262
5–8	362.7957 ± 201.9709	15.37143 ± 9.4688
	*t* = 1.9972 *p* = 0.1250	*t* = 0.1221 *p* = 0.4522

The average BMI was 30.05 ± 4.89 kg/m ^2^ , with nine patients having a BMI of less than 30 kg/m ^2^ and 7 of them had a BMI greater than 30 kg/m ^2^ . The mean volume difference at 12 months post-LVA was 485.65 ± 41.108 and 400.88 ± 31.487 mL ( *t* = 0.6443) with a percentage difference of 8.41 and 15.57%, respectively. We did not find any statistically significant difference in volume reductions from baseline ( Table 3 ) at the 12-month follow-up period between the two groups ( *p* = 0.2648 and 0.2954 for volume and percentage).

**Table 3:** Relation between BMI and volume reductions at 12 months post-LVA

BMI	*N*	Volume difference at 12 mo (mL)	Volume difference at 12 mo (%)
< 30 kg/m ^2^	9	485.65 ± 41.108	8.41 ± 5.3438
> 30 kg/m ^2^	7	400.88 ± 31.487	15.57 ± 1.6263
		*t* = 0.6443 *p* = 0.2648	*t* = 0.5500 *p* = 0.2954

Abbreviations: BMI, body mass index; LVA, lympho **-** venous anastomosis.

The average time duration for the presence of lymphedema was 19.66 ± 4.67 months. The duration of lymphedema was less than 18 months in 5 patients and more than 18 months in 11 patients. The mean volume difference at 12 months post-LVA was 374.84 ± 150.3 and 482.106 ± 291.994 mL ( *t* = 0.7662), with a percentage volume reduction of 12.47 and 16.42%, respectively. We did not find any statistically significant difference in volume reductions ( Table 4 ) from baseline at the 12-month follow-up period between the two groups ( *p* = 0.2281 and 0.1943 for volume and percentage).

**Table 4:** Relation between lymphedema duration and volume reductions at 12 months post lympho-venous anastomosis (LVA)

Duration of lymphedema	*N*	Volume difference at 12 mo (mL)	Volume difference at 12 mo (%)
< 18 mo	5	374.84 ± 150.3	12.47 ± 5.2327
> 18 mo	11	482.106 ± 291.994	16.42 ± 9.1555
		*t* = –0.7662 *p* = 0.2281	*t* = –0.8896 *p* = 0.1943

The efficacy of the procedure was assessed from the results of the volumetric analysis in volume difference between two limbs and percentage difference.

## Efficacy

**Fig. 4:**
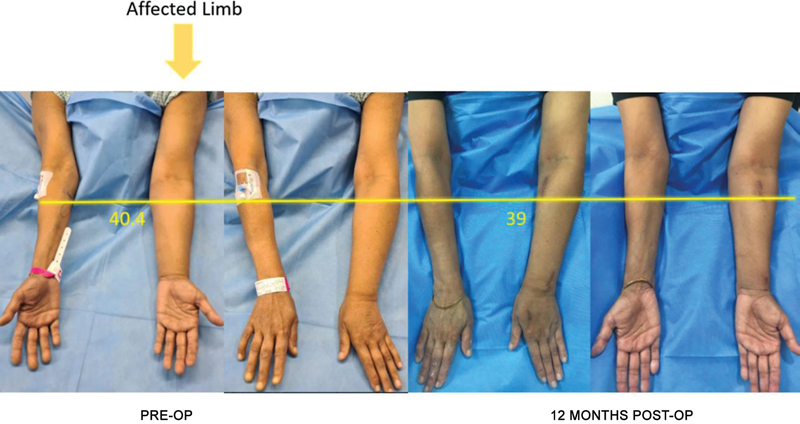
Preoperative and 12-month postoperative images with volumetric marking at 4 cm below the cubital crease.

The preoperative mean relative difference in limb circumference volume( Table 5 ) was 804.41 ± 472.01 mL. This was reduced to 471.81 ± 292.216 mL at 6 months of follow-up ( *t* = –6.6323; *p* ≤ 0.00001) and 448.58 ± 255.93 mL ( *t* = –5.5295; *p* = 0.00006) at 12 months of follow-up. At 18 months of follow-up, the mean difference in limb circumference was 445.25 ± 345.78 mL ( *t* = –6.8957; *p* ≤ 0.00001). The percentage volume reductions at 6, 12, and 18 months of follow-ups were 51.29, 54.83, and 55.23%, respectively. The absolute volume reduction of the affected limb was 2,144.1 ± 244, 1,864.1 ± 209.97, and 1,786.4 ± 208.92 mL at 6, 12, and 18 months of follow-ups, respectively, from 2,641 ± 422.56 mL preoperatively ( Figs. 4 5 6 ).

**Table 5:** Efficacy of secondary lympho-venous anastomosis (LVA) based on volumetric analysis

Time	Relative volume difference (mL)		Relative volume difference (%)		Absolute volume difference (mL)	
Preoperative	804.41 ± 472.01		32.5 ± 14.2082		2,641 ± 422.56	
Postoperative (6 mo)	471.81 ± 292.216	*t* = –6.6323 *p* ≤ 0.00001	15.83 ± 0.087	*t* = –7.2227 *p* ≤ 0.00001	2,144.1 ± 244	*t* = –25.1147 *p* ≤ 0.00001
Postoperative (12 mo)	448.58 ± 255.93	*t* = –5.5295 *p* = 0.00006	14.68 ± 9.09	*t* = –6.9821 *p* ≤ 0.00001	1,864.1 ± 209.97	*t* = –28.477 *p* ≤ 0.00001
Postoperative (18 mo)	445.25 ± 345.78	*t* = –6.8957 *p* ≤ 0.00001	14.55 ± 8.06	*t* = –7.5379 *p* ≤ 0.00001	1,786.4 ± 208.92	*t* = –28.8195 *p* ≤ 0.00001

**Fig. 5:**
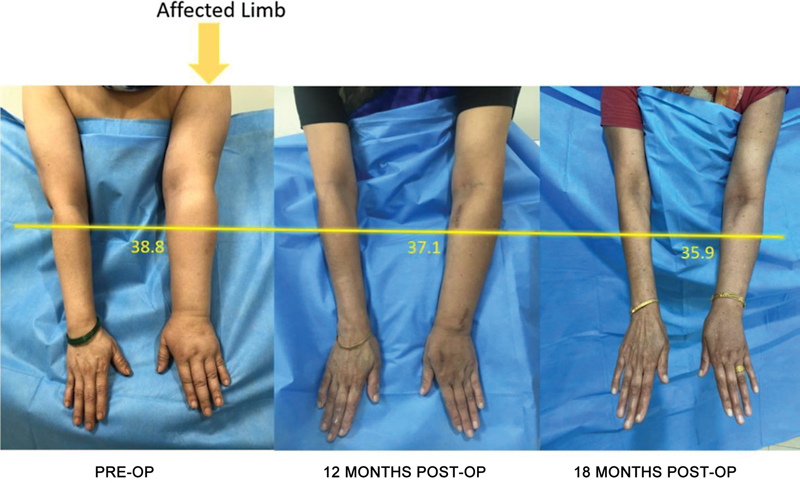
Preoperative and 12- and 18-month postoperative images with volumetric marking at 4 cm below the cubital crease.

**Fig. 6:**
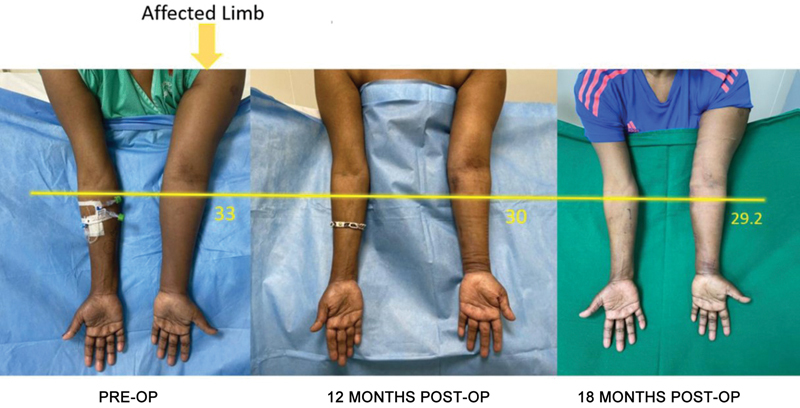
Preoperative and 12- and 18-month postoperative images with volumetric marking at 4 cm below the cubital crease.

The ILLS system was devised in our institute for the Indian population with lymphedema to know the quality of life. The scoring system included physical, mental, social wellness, and activities of daily living modules, which were filled by patients both pre- and postoperatively ( Table 6 ). All patients were able to answer the questionnaire. The average of the scoring system indicated moderate to very severe problems preoperatively in the pain and heaviness subunit of the physical module, all subunits of the social module, and the activities of daily living module. The highest scores of 4.37 and 4.75 were obtained in blouse sleeve size and bangle size discrepancies, respectively, compared to other categories preoperatively. Postoperatively, the average scores reported in each of the domains ranged from minimal to mild problems in terms of severity. An analysis done using deductive thematic analysis and paired *t* -test to analyze the preoperative and post operative outcomes showed a statistically significant reduction in problems in each of the domains individually except patient-reported sadness and anxiety. The mean total ILLS scores were 31.35 and 13.66 pre- and postoperatively, respectively, showing statistically significant differences, with a *p* -value of 0.00023.

**Table 6:** Indian limb lymphedema scoring system (ILLS) disability, activity restrictions, and social limitations in the Indian population of the upper and lower limb lymphedema

ILLS domains	Questions	Average pre-op score (0–5)	Average post-op score (0–5)	*t* -Value	*p* -Value
Physical well-being	Pain and heaviness	4.06	1.87	–16.0873	< 0.00001
	Tightness/skin changes	1.62	1.13	–3.1622	0.0064
	Incidence of lymphangitis	2.81	1.37	–7.0643	< 0.00001
Activities of daily living	Household activities	3.75	1.06	–13.5526	< 0.00001
	Dependency on family	3.50	1.37	–6.7551	< 0.00001
Mental well-being	Lack of self-confidence	1.92	0.95	–4.8692	0.0002
	Sad/anxious	1.25	1.18	–1	0.33
Social well-being	Do your job	3.37	1.31	–10.6878	< 0.00001
	Blouse sleeve size discrepancy	4.37	1.81	–14.0905	< 0.00001
	Bangles size discrepancy	4.75	2.37	–10.7337	< 0.00001
Total score		31.35	13.66	–5.8985	0.00023

Note: 0 = not at all; 1 = minimal; 2 = mild; 3 = moderate; 4 = severe; 5 = very severe.

## Satisfaction Module

In general, among various subunits of the satisfaction module ( Table 7 ), patients reported almost similar satisfaction in all modules analyzed by the mean outcomes, which were included within two standard deviations. Their satisfaction with information provided to them regarding the surgery and postoperative care was the highest.

**Table 7:** Satisfaction scoring

Characteristic	Score (0–10)	Standard deviation
Satisfaction with limb (postoperative outcome)	7.18	1.17
Satisfaction with scar	7.12	1.54
Satisfaction with surgeon	8.13	1.02
Satisfaction with information	8.75	0.44
Satisfaction with medical staff	7.93	0.77
Satisfaction with other staff	7.43	1.03

## Discussion

This study includes efficacy, patient satisfaction, and quality of life of patients undergoing secondary LVA after oncological resection in 16 patients.

Microsurgery techniques allow us to create a shunt for lymphatic flow, which is obstructed following lymph node dissection and radiation, into the venous circulation and help in decompression of the limb.

The participants in this study ( *n* = 16) had many features representative of the base population but underwent lymph node clearance and radiotherapy, which is a predisposing factor for lymphedema.

The maximal percentage volume reduction noted in our study was at 12 months of follow-up volumetry, which was 54.83% ( *p* ≤ 0.00001). The results were comparable to those of Campisi et al who observed over 75% edema reduction in 73% ( *n* = 616) patients and over 50% volume reductions in 24% ( *n* = 202) of patients. In this study, secondary LVA has proven to be beneficial, especially in early stages in which LVA (by reestablishing preferential lymphatic drainage pathways in the affected limb) provides very good results, and even near complete healing. 11 Similarly, a study by van Cruchten and Nieuborg in 1995 ( *n* = 1,400) showed percentage volume reductions in limbs as 81, 69, 57, and 31% in stages 1, 2, 3, and 4, respectively. 12 The results in our study are superior when compared to the study conducted by Damstra et al in 2008 on 10 patients with lymphedema, which showed only 16 and 9% volume difference in the limbs at 3 and 12 months of follow-up after secondary LVA. 13

The mean total ILLS scores were 31.35 and 13.66 pre- and postoperatively, respectively, showing statistically significant differences ( *p* = 0.00023). The average score for pain and heaviness preoperatively was the highest in the physical domain with subsequent improvement in the postoperative period ( *p* ≤ 0.00001). There was a substantial improvement in activities of daily living after secondary LVA with *p* ≤ 0.00001. Improvement was noted in the confidence level of patients with limb lymphedema ( *p* = 0.0002), but the sad/anxious mood of the patients failed to improve after surgery ( *p* = 0.33), which may indicate cultural stigma and beliefs. The highest social well-being scores were recorded in discrepancies of blouse sleeve size and bangle size, with subsequent improvement in both parameters following surgery ( *p* ≤ 0.00001). However, none of the parameters showed complete remission, which suggests involvement of other factors like hypertrophied soft tissue and lack of adherence to other supportive measures like compression and limb elevation.

The approach to evaluate the quality of life in our study focused on the serious impact that lymphedema can have on the patient's life in India.

Treatment is usually aimed at reducing the size and volume of the limb and edema reduction is measured to assess the outcome of treatment. The findings from this study suggest that for most women, treatment should be aimed at decreasing pain, activity limitations, and participation restrictions, and the success of treatment should be measured by the women's ability to participate in various activities. 14

There are several limitations of our study. First, the screening questionnaire we employed for this study had not yet undergone validity testing, even though the items' substance was derived from the questionnaires used in other studies. The study's statistics provide early support for the reliability of this screening questionnaire. Second, a larger sample size with a longer follow-up period and randomization would provide further information about treatment outcome.

## Conclusion

LVA significantly improves the quality of life of the patients and has been shown to reduce limb circumference discrepancies to a great extent, stabilizing at 18 months of follow-up. In addition, the number of anastomoses, the patient's BMI, and lymphedema duration did not affect volume reduction or quality of life. The quality of life questionnaire devised for the Indian population that gives the ILLS score has completed phase 1 of the trial and is currently undergoing phase 2 of development.
